# A High-Sensitivity Fiber Optic Soil Moisture Sensor Based on D-Shaped Fiber and Tin Oxide Thin Film Coatings

**DOI:** 10.3390/s24237474

**Published:** 2024-11-23

**Authors:** Chuen-Lin Tien, Hsi-Fu Shih, Jia-Kai Tien, Ching-Chiun Wang

**Affiliations:** 1 Department of Electrical Engineering, Feng Chia University, Taichung 40724, Taiwan; 2 Department of Mechanical Engineering, National Chung Hsing University, Taichung 402202, Taiwan; hfshih@nchu.edu.tw; 3 Graduate Institute of Library & Information Science, National Chung Hsing University, Taichung 402202, Taiwan; tienalex020290@gmail.com; 4 Mechanical and Systems Research Laboratory, Industrial Technology Research Institute, Hsinchu 310401, Taiwan; juin0306@itri.org.tw

**Keywords:** D-shaped fiber, thin film, lossy mode resonance, soil moisture sensor

## Abstract

We present a high-sensitivity fiber optic soil moisture sensor based on side-polished multimode fibers and lossy mode resonance (LMR). The multimode fibers (MMFs), after side-polishing to form a D-shaped structure, are coated with a single-layer SnO_2_ thin film by electron beam evaporation with ion-assisted deposition technology. The LMR effect can be obtained when the refractive index of the thin film is positive and greater than its extinction coefficient and the real part of the external medium permittivity. The D-shaped fiber optic soil moisture sensor was placed in soil, allowing moisture to penetrate into the thin film microstructure, and it observed the resonance wavelength shift in LMR spectra to measure the relative humidity change in soil. Meanwhile, an Arduino electronic soil moisture sensing module was used as the experimental control group, with soil relative humidity ranging from 10%RH to 90%RH. We found that the D-shaped fiber with a residual thickness of 93 μm and SnO_2_ thin film thickness of 450 nm had a maximum sensitivity of 2.29 nm/%RH, with relative humidity varying from 10%RH to 90%RH. The D-shaped fiber also demonstrates a fast response time and good reproducibility.

## 1. Introduction

Due to the rapid development of science and technology, optical fiber sensors are very important in various fields of industry, such as the following: biomedical sensing, engineering monitoring, pH value measurement, pressure sensing, liquid refractive index sensing, etc. Using optical fibers as a sensing medium could be a future trend. When it comes to soil moisture sensors, there are many soil moisture meters on the market that can be inserted into soil to detect moisture. While many electronic soil moisture sensors exist, they are prone to corrosion and failure over time. Fiber optic sensors can overcome these problems because they are passive sensors which are not susceptible to electromagnetic signal interference and can adapt to electrical, chemical, humid, and other environments. In addition, fiber optic sensors are small in size and light in weight, making them easier to be placed in small spaces for long-distance network monitoring. Soil moisture sensors are crucial for precision agriculture, optimizing water use, and increasing crop yields. Soil moisture sensors are widely used in agricultural and environmental applications to measure the water content of soil. There are several types of soil moisture sensors available on the market. Most of them are electrodynamic sensors, including time domain reflectometers (TDRs) [1,2], time domain transmission sensors [3,4], and capacitive sensors [5]. Time domain reflectometry sensors use electromagnetic waves to measure soil moisture content. Time domain transmission (TDT) sensors cost less compared to TDR sensors. However, TDR and TDT are not the solution to all soil water content sensing problems. Capacitance sensors measure soil moisture content by determining the dielectric constant of soil. Many capacitance sensors are sensitive to interference from bulk soil electrical conductivity. Although the use of these sensors is feasible, they require power supplies and suffer from signal attenuation in cables, making them difficult to implement across hundreds of acres of farmland.

Up-to-date optical fiber sensors (OFSs) based on various optical fiber structures have been reported, starting from the most common standard silica glass single-mode fiber (SMF) and multimode fiber (MMF) to highly structured fibers made of special materials [6,7]. For high-sensitivity fiber optic sensor applications, the light guided in the fiber must interact with an external medium, such as an evanescent field. As the optical properties of the external medium change, this has an impact on guiding conditions in the optical fiber [8]. To obtain the interactions between the light guided in the fiber and the external medium, the electromagnetic field in the optical fiber needs to reach the medium [9]. Many methods have been applied for cladding removal purposes, resulting in the development of a D-shaped fiber section that can be used as the active part of the optical fiber sensor [10]. For example, optical fiber shapes can be fabricated and changed by a motor-driven polishing wheel [11], embedding fibers in silicon V grooves [12], chemical etching [13], hot melt drawing [14], and the electromechanical side-polishing method [15], but chemical etching and hot melt drawing will make the fiber structure fragile. Other methods can easily cause fiber breakage, so we use a motor-driven electromechanical side-polishing technique to control the residual thickness of optical fibers.

Surface plasmon resonance (SPR) and lossy mode resonance (LMR) are two major electromagnetic resonances described in the literature [16]. In general, an SPR phenomenon can be obtained when the real part of the complex permittivity of the thin film coating is negative and greater in magnitude than its imaginary part and the real part of the external medium permittivity. On the contrary, the conditions for LMR generation are that the real part of the complex permittivity of the thin film coating is positive and greater than its imaginary part and the real part of the external medium permittivity. These conditions imply that only conductive materials can be used for SPR generation, while a wider range of materials such as dielectrics, semiconductors, or polymers can be used for LMR-supporting thin films. It should be noted that the LMR sensor technique seems to be cheaper than the SPR-based one because the coating materials used in SPR sensor manufacturing are relatively expensive, such as gold, which is most commonly used. For fiber optic sensors based on the LMR effect, the main parameters to be considered are film thickness and the thin film refractive index. Ozcariz et al. [17] reported that when the film thickness increases, the number of resonant wavelengths increases as well. The resonant wavelength position also shifts to longer wavelengths. Verma et al. [18] also mentioned that lossy mode resonance occurs when a lossy mode starts to be guided in a thin film, which leads to an increase in the imaginary part of the effective refractive index of the guided mode in the optical fiber. Therefore, the LMR condition indicates that the real part of the dielectric constant of the thin film material must be positive, and its magnitude must be greater than its own imaginary part and the dielectric constants of the optical fiber and surrounding medium. In 2011, Zamarreno and Hernaez et al. [19] proposed to use titanium dioxide (TiO_2_) and polymer (PSS) as the thin film material on the optical fiber to make a fiber optic relative humidity sensor based on lossy mode resonance. They used multimode fibers and removed a small section of the cladding as a sensing area and then adopted layer-by-layer deposition technology (LBL) to deposit 40 pairs of a double-layer polymerization structure (film thickness of about 1.3 μm). When the polymer PSS is combined with moisture in the external environment to expand, the effective refractive index will increase, thereby indirectly affecting the refractive index change in the TiO_2_ thin film.

In 2019, Hernaez et al. [20] reported on an LMR-based humidity optical fiber sensor with graphene oxide nanostructured coatings. The LMR-based optical fiber sensing scheme consisted of a multimode optical fiber with a layer of SnO_2_ directly sputtered onto the core, which acts as an LMR-supporting coating, and a graphene-based thin film deposited on top, acting as the sensitive coating. The average sensitivity across the complete range of study (20–90%RH) reaches 0.612 nm/%RH. However, the sensitivity of the sensor is not constant over the whole interval of relative humidity. Hromadka et al. [21] conducted multiparameter temperature and relative humidity monitoring by using a long-period grating (LPG) array to detect air delivered during mechanical ventilation. The LPG array enabled the measurement of temperature and RH changes with a sensitivity of 0.46 ± 0.01 nm/°C and 0.53 nm/%RH, which was related to the nonbreathing and breathing modes at two different respiratory rates of the mechanical ventilator typically used in clinical settings. In 2021, Wang et al. [22] proposed a polymer optical fiber Bragg grating (POFBG) probe for measuring soil moisture. The sensor probe was embedded in soils with different gravimetric soil moisture contents (SMCs), ranging from 0% to 40%. The maximal sensitivity was calculated to be 0.018 nm/% at 20 °C. In 2022, Leon et al. [23] reported on a method for measuring soil water content by using a smart optical fiber probe judiciously combined with a nanoporous Al_2_O_3_ ceramic disk, which was used as the sensing material. The results showed its capability to detect soil water content variation in the 0–35% range of volumetric water content (VWC) with a sensitivity of 2.3%/%VWC and a VWC resolution lower than 1%. In the same year, Raju et al. [24] proposed an optical humidity sensor based on a Bragg grating coated with polyimide that can be deployed in harsh environments such as sewer structures. The results showed that the Bragg wavelength changes linearly over a humidity range of 11% to 97% with a sensitivity of 2.0 pm/%RH, which is optimal for deployment in sewer environments. Xia et al. [25] proposed a fiber optic Fabry–Perot (FP)-based relative humidity (RH) sensor. They used Poly (N-isopropylacrylamide) (PNIPAM) hydrogel to connect two ends of a single-mode optical fiber (SMF) to formulate a PNIPAM FP cavity. Water vapor was absorbed by the PNIPAM hydrogel, which altered both the refractive index (RI) and dimension of the PNIPAM FP cavity, resulting in a change in output resonance intensity in the reflective spectrum. The measured RH sensitivity was 1.634 nm/%RH within the RH range from 45 to 75%RH with a good linearity of 0.9897. In addition, Liu et al. [26] reported an optical fiber probe functionalized with “cotton-like” gold silica nanostructures for relative humidity (RH) monitoring. The sensor response utilized the localized surface plasmon resonance (LSPR) of self-assembled nanostructures, which are composed of gold nanospheres (40 nm) surrounded by one layer of poly (allylamine hydrochloride) and hydrophilic silica nanoparticles (10–20 nm) on the end-facet of an optical fiber via a wavelength shift in the reflected light. The plasmonic hybridization mode sensor exhibited a high linear regression from 60%RH to 90%RH with a sensitivity of 0.63 nm/%RH.

In the literature, the term ‘humidity’ refers to water in gaseous form, but it is commonly used to refer to expressions related to the characteristics of water vapor. There are various terms related to such water vapor measurements in the field of measurements. In addition, the term ‘moisture’ is often interchangeable with ‘humidity’, although the actual definition of ‘moisture’ refers to liquid water that may exist in solid materials [27]. Relative humidity (RH) sensors can enable us to determine the water vapor activity in soil, which is equivalent to the moisture content of liquid soil water [28]. Since we want to measure soil moisture, we must use moisture-sensitive materials when selecting materials to be coated on the optical fiber polishing surface so that the film can effectively absorb moisture, allowing moisture to penetrate the microstructure of the thin film, thus increasing the refractive index.

All of the techniques discussed so far, covering different approaches to designing various architectures to provide humidity sensing, have unique advantages and disadvantages, but none of them can simultaneously satisfy the requirements of accuracy, cost, ease of operation and maintenance, background interference, and operation, such as environment and remote operation. In order to detect the relative humidity in soil, this study proposes and manufactures a new LMR-based fiber optic humidity sensor. D-shaped optical fibers combined with the thin film coating technique are used to fabricate a new fiber optic soil moisture sensor. Since the fiber optic soil moisture sensors have a significant impact on smart agriculture by providing real-time information about soil water contents, this information could be used to optimize irrigation, reduce water usage, and increase crop yields. Fiber optic soil moisture sensors can also be used to prevent diseases caused by excessive moisture or dryness in soil. By providing accurate, real-time information about the soil moisture content, farmers can take appropriate measures to prevent diseases caused by unfavorable moisture conditions.

Therefore, we focus on the design and fabrication of fiber optic soil moisture sensors to research and develop a high-sensitivity optical thin film sensor that can be practically applied to soil moisture measurement. We use a tin dioxide (SnO_2_) thin film as the sensitive covering material, and it also has good chemical stability. Based on the excellent durability and various advantages of the above-mentioned optical fibers, combined with current thin film sputtering and evaporation technologies, a thin film with good uniformity, repeatability, and durability can be obtained during coating. This combination of fiber optic and process technology can be applied in various fields, including the production of various fiber optic sensors.

## 2. Materials and Methods

In this study, a high-sensitivity fiber optic soil moisture sensor is proposed. The fabrication of D-shaped multimode fibers and the design of an LMR fiber optic soil moisture sensor are described as follows.

### 2.1. Fabrication of D-Shaped Multimode Fibers

A homemade fiber optic side-polishing machine fabricated the proposed D-shaped multimode fibers (MMFs). The outermost protective layer (jacket) must be stripped off before polishing. When the optical fibers perform side-polishing, they are fixed on a special fixture and set on the side-polishing machine. The side grinding of optical fibers is mainly carried out through the side-polishing skin and polishing fluid on the machine roller. If the pulling force and the contact area between the optical fiber and the machine drum are matched, the side-polishing effect can be enhanced, and the polishing time can be reduced. The rotation speed of the polishing machine should not be too slow; otherwise, the ideal polishing effect cannot be achieved. If the polishing machine’s rotation speed is too fast, the optical fiber will shake violently and cause breakage. In addition, due to the precipitation of polishing powder (CeO_2_), the concentration distribution of the polishing fluid must be controlled to be very uniform.

**Figure 1:**
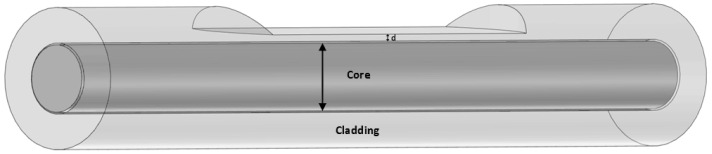
Schematic diagram of D-shaped multimode fiber structure.

**Figure 2:**
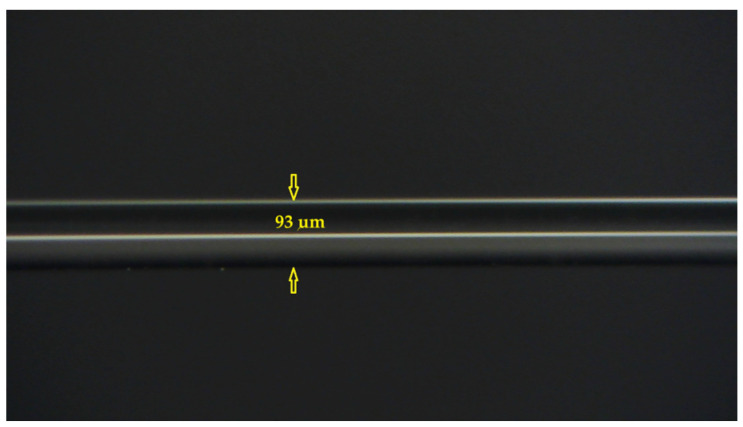
Microscopy image of D-shaped multimode optical fiber after side-polishing.

The multimode fiber (MMF) side-polishing process was equipped with a real-time optical monitoring system to control the residual thickness of the optical multimode fibers. The purpose of polishing the cladding part of the optical fiber into a D-shaped fiber is to make the optical signal passing through the sensing part of the fiber closer to the external environment, thereby promoting the interaction with the environmental medium and changing the optical properties in the fiber core, such as the disappearance of total reflection conditions, thus affecting light intensity, polarization, and phase. In this work, D-shaped multimode optical fibers were polished through a motor-driven electromechanical side-polishing method [15]. This side-polishing method can greatly reduce polishing time and produce any uniform polishing surface and polishing length. By slightly adjusting the polishing wheel speed to increase or decrease the fiber contact-point tension, the length of the polished surface can be changed to the desired length of the D-shaped fiber sensing area. The creation of the structure of the D-shaped optical fiber is completed by side grinding and side-polishing. A three-dimensional schematic diagram of the D-shaped multimode optical fiber is shown in Figure 1. The residual thickness of the MMF after polishing is in line with expectations. A microscopic image of the side-polished surface of the D-shaped MMF is shown in Figure 2.

### 2.2. Sensor Design Based on Lossy Mode Resonance

As a moisture-sensitive material, tin dioxide has good physical adsorption to water vapor and is very suitable for measuring humidity. The soil moisture sensing area of the completed D-shaped fiber sensor is buried in soil. As soil moisture increases, the amount of water vapor adsorption increases, resulting in a change in the effective refractive index of the thin film. The proposed sensor based on tin oxide (SnO_2_)-coated optical fibers can be used for the optical monitoring of relative humidity changes. When the optical constants and thickness of the SnO_2_ thin film meet the phase-matching conditions, the lossy mode resonance effect can be generated and used to monitor the optical refractive index of analytes near the film surface. Therefore, this study uses a SnO_2_-coated D-shaped MMF to fabricate a soil moisture sensor, in which the optical effect is based on lossy mode resonance (LMR) rather than the well-known surface plasmon resonance (SPR). The residual thickness of the D-shaped fiber is 93 μm.

**Figure 3:**
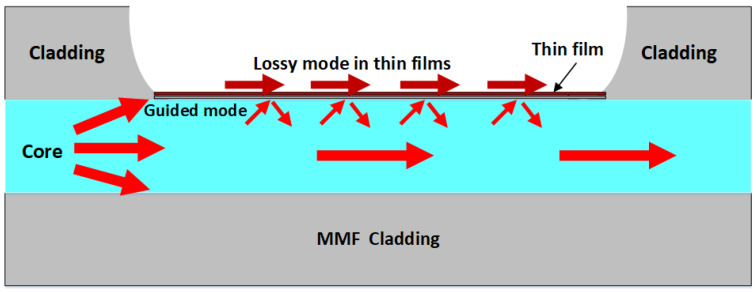
Schematic diagram of LMR phase-matching condition.

Many optical fiber sensors are based on the LMR sensing mechanism [29,30,31,32,33]. The occurrence of the LMR phenomenon is due to the modal coupling between the guided mode of the core and the loss mode in the thin film. The guided mode of the core will penetrate the thin film, and then when the phase-matching condition is satisfied, it will become mode-guided in the film. The schematic diagram of the LMR phase-matching condition is shown in Figure 3. Due to the formation conditions of the lossy mode resonance sensor, modal guidance can be formed as long as specific phase conditions between the guided mode and the lossy mode are met. When the refractive index of the surrounding medium changes, the effective refractive index of light propagation inside the thin film will also change, thereby changing the phase-matching condition and causing a change in the resonance wavelength.

When lossy mode resonance occurs, there will be one or more attenuation dips in the spectrum. The sensing principle of the LMR-based soil moisture sensor is explained as follows. The thin film coating area of the D-shaped fiber serves as the sensing area. When the soil moisture changes, the effective refractive index for light propagating within the thin film overlay also changes. Therefore, the resonance wavelength also changes due to changes in the phase-matching conditions. Wang et al. [34] mentioned that the change in the refractive index of the external medium will lead to a change in the phase-matching conditions between the core mode and the loss mode, resulting in different loss spectra. Therefore, the change in the refractive index of the external medium to be measured can be effectively detected by the shift in the measured resonance wavelength. This view is consistent with our previously published results [35].

Lossy mode resonance (LMR) is a relatively new physical optical phenomenon proposed over the past decade. The use of LMR fiber optic sensors provides a new method for improving sensing capability. LMR fiber optic sensors have various structures, such as D-shaped, cladding, fiber tip, U-shaped, and tapered fiber structures. The main applications of LMR sensors include liquid refractive sensors, temperature and humidity sensors, gas sensors, and biosensors [36,37,38,39]. Unlike SPR with only one resonant valley, LMR spectra generate multiple resonance dips and can also be tuned by changing the thickness of the coating film without any modification to the fiber geometry. This means that when measuring a single parameter, the accuracy of the measurement can be ensured by obtaining information from multiple resonance dips, while when measuring multiple parameters, different parameter information can be obtained from different resonance dips, which helps with signal demodulation. This feature makes the LMR technique more suitable for manufacturing multimodal sensors with higher sensitivity and better accuracy.

## 3. Experimental Details

The optical fiber soil moisture sensor designed and developed in this study can lead to more effective water resource utilization, higher crop yields, and improved environmental sustainability when applied in smart agriculture. Due to problems such as the aging of the agricultural population and the lack of labor, the development of agricultural farming technology is moving towards the goals of automation and refinement. In the future, smart sensing technology will be introduced into agricultural production, and smart sensors will be used to collect crop, environmental, and field operation data. Through the application of big data analysis and prediction models, effective agricultural land management can be carried out, and a high-efficiency management model can be attained. In the past, the general farming method for farmers was to observe soil moisture, and most farmers did not use sensors to detect soil moisture. In the research process, we understand the importance and issues of developing smart agriculture. The proposed soil moisture sensing system can measure the relative humidity of soil in real-time. In terms of future applications, the collected and captured sensor data (such as temperature, soil moisture, etc.) can also be uploaded to a cloud database. Transforming data into useful information for agricultural operations can provide a reference for business decision-making, such as that in crop production and marketing planning, production management, and customer service for farm managers; assist in the intelligent monitoring of production and marketing processes; reduce the burden of farm operations; and decrease labor demand.

In order to achieve this goal, this study designed and fabricated a highly sensitive soil moisture sensor based on the LMR effect. The experimental method is described below.

### 3.1. D-Shaped Fiber Sensor Coated with SnO_2_ Thin Film

**Figure 4:**
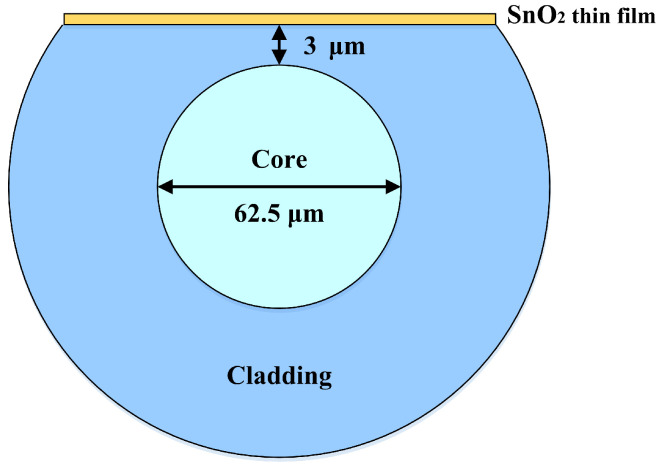
The proposed D-shaped MMF coated with a SnO_2_ thin film.

In the fabrication of side-polishing D-shaped fibers with different residual thicknesses (approximately 90–95 μm), multimode fibers (MMFs) were polished using a homemade fiber-polishing system. The MMF cladding was removed until a strong evanescent wave was produced. The surface roughness of the side-polishing D-shaped fibers has to be reduced to avoid scattering loss. The distance between the side-polishing flattened surface and core region is 3 μm. The remaining thickness of the D-shaped MMF is about 93 μm. The polished fiber length of the MMF is 30 mm for the proposed sensing device. A schematic of our proposed LMR sensing device based on the D-shaped MMF is illustrated in Figure 4.

Tin oxide (SnO_2_) is an n-type semiconductor material with excellent physical properties, chemical stability, and low cost. In addition, SnO_2_ coatings are relatively hard and have good abrasion and scratch resistance [40]. To develop highly sensitive soil moisture sensors, multimode optical fibers need to be combined with side-polishing and thin film coating technologies. After completing the side-polishing process to form D-shaped fibers, the residual thicknesses of the side-polished fibers are measured using an optical image microscope with analysis software. The side-polishing surfaces of the D-shaped fibers are placed in an electron beam evaporation coating machine. Tin oxide thin film is a humidity-sensitive material with a high refractive index and good chemical stability, and it has a high adsorption capacity for water vapor. Therefore, this research focuses on the SnO_2_ thin film used as an optical fiber soil moisture sensor.

**Figure 5:**
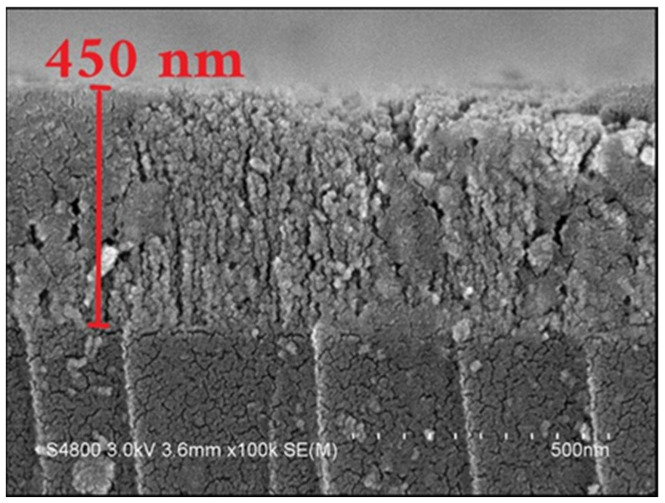
Cross-sectional view of SEM image of SnO_2_ thin film.

Tin oxide thin films with a thickness of 450 nm were deposited on D-shaped multimode fibers and B270 glass by electron beam evaporation associated with the ion beam-assisted deposition technique. An optical-grade SnO_2_ coating material with a purity of 99.95% was used as the target material for depositing the thin films. Film deposition was realized by employing a 4 kW electron beam gun with sweep and automatic emission control. The base chamber pressure before deposition was 5.0 × 10^−4^ Pa to deposit films with good purity. The working pressure was set at 2.0 × 10^−2^ Pa, and the ion source gas was argon gas. Oxygen gas with 99.99% purity was supplied into the chamber using a mass flow controller for SnO_2_ thin film deposition. The substrate holder (dome-type) was rotated at 24 revolutions per minute continuously to ensure a good thickness uniformity of the thin films. The film thickness and the deposition rate were controlled using a quartz crystal sensor and an optical transmission spectrum monitoring system, respectively. The deposition rate of the thin films was 1.0 nm/s. The D-shaped fibers, silicon wafer, and glass substrates were heated to 150 °C by a radiative heater from the backside of the substrate holder for 30 min before deposition to obtain a uniform temperature over the substrate and to provide good adhesion to the SnO_2_ film with a substrate. The thickness of the SnO_2_ thin film deposited on a D-shaped MMF was measured by using high-resolution scanning electron microscopy (SEM, Hitachi S3000, Hitachi Co., Ltd., Tokyo, Japan). The cross-section morphology and microstructure of SnO_2_ thin film were also investigated using SEM images. Figure 5 shows the SEM image of a 450 nm thick SnO_2_ thin film deposited on a silicon wafer. The SEM image shows the columnar structure and void formation during the growth process of the SnO_2_ thin film. The electron beam-evaporated SnO_2_ thin films are amorphous. The research results revealed the influence of experimental deposition conditions on the properties of SnO_2_ thin films. Many studies have shown that the deposition conditions of thin films can affect their microstructure, surface morphology, optical and electrical properties [41,42], etc. 

### 3.2. Experiment on LMR Soil Moisture Sensor

**Figure 6:**
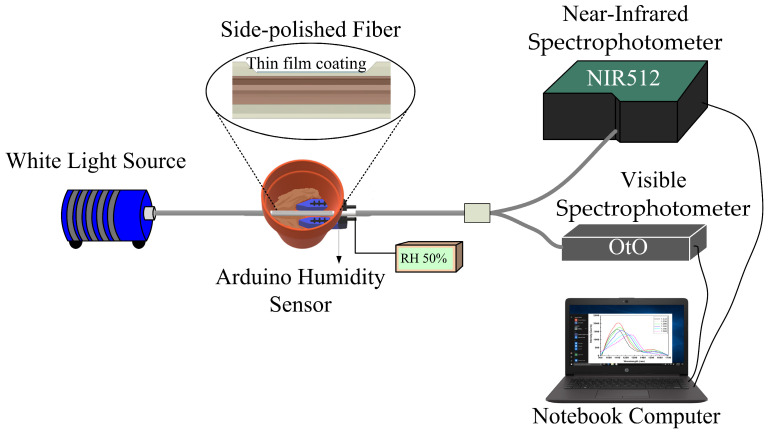
Experimental setup of fiber soil moisture sensor.

After thin film deposition, the transmission spectrum of the coated MMF was measured and analyzed. Then, we buried the D-shaped LMR fiber sensor and an Arduino electronic soil moisture device in soil to measure soil moisture and to compare the transmission spectra under different relative humidity percentages. The setup of the soil moisture sensing experiment in the laboratory is shown in Figure 6. All measurements were performed at a constant room temperature of 25 °C. One end of the fiber was connected to a halogen light source. The transmission spectra were recorded by two kinds of spectrophotometers, and their transmission spectra in two different wavelength ranges (wavelengths 400–1000 nm and 900–1700 nm) were displayed on a personal computer. One was recorded by a near-infrared (NIR) spectrophotometer (NIRQuest512, Ocean Optics, Orlando, FL, USA); the other was recorded by a visible band spectrophotometer (Oto Photonics, Hsinchu City, Taiwan). The corresponding spectra of soil moisture from RH10% to 90% were recorded. Due to the wide wavelength range of halogen light sources, they can simultaneously receive visible and near-infrared light, rather than a single-wavelength spectrum, which is beneficial for us to obtain resonant wavelength shifts based on the lossy mode resonance effect. Meanwhile, the Arduino electronic soil moisture sensing device was used as the experimental control group. The humidity sensing range set in the experiment was from 10%RH to 90%RH. It can be clearly observed that the LMR wavelength region was generated in the near-infrared light region, and as the soil moisture (in %RH) increased, there was an obvious LMR wavelength shift phenomenon, but the resonance wavelength change in the visible light transmission spectrum range is small. This was based on the observed shift in the spectral LMR wavelength under different humidity conditions. Finally, the sensitivity of the D-shaped fiber optic soil humidity sensor was determined by the curve fitting method.

## 4. Results and Discussion

### 4.1. Sensitivity

Using the LMR method as a sensing mechanism can facilitate the fabrication of different soil moisture sensors. The sensitivity of LMR-based fiber optic sensors was analyzed by using the curve fitting method. The sensitivity (*S*) of the LMR soil moisture sensor is defined as the following formula:

*S* = Δ*λ*/ΔRH, (1)

where Δ*λ* is the resonance wavelength shift, and ΔRH is the variation in soil moisture in terms of relative humidity.

**Figure 7:**
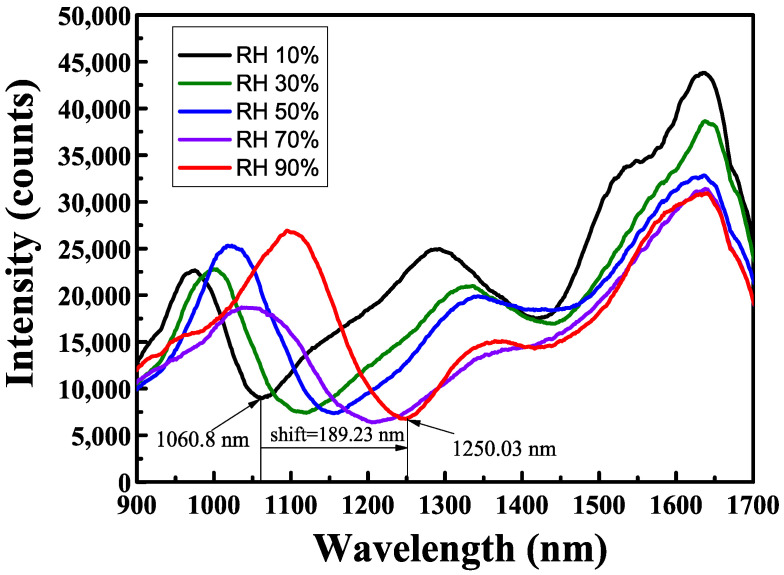
Spectra of soil moisture sensor with single-layer SnO_2_ thin film.

Figure 7 shows the transmission spectrum of a single-layer SnO_2_ film coated on the D-shaped multimode fiber for the soil moisture sensing experiment. The location of LMR dips in the transmission spectrum depends on the film thickness of the SnO_2_ coatings. A thicker film will produce resonance at a longer wavelength. In the thin film coating experiment, a B270 glass plate was placed next to the D-shaped fibers to act as a probe. The deposited film thickness can be determined. The results are presented in Figure 7, where the transmission spectra vary with the soil moisture contents (10–90%RH). The *y*-axis in Figure 7 represents the intensity of transmitted light, using the number of photons (counts) detected by the instrument itself as the unit. Several attenuation dips can be observed in the graphic. These dips are associated with a coupling between the waveguide mode and lossy mode in the SnO_2_ thin film that occurs at a particular resonance wavelength depending on the thickness of the SnO_2_ thin film.

**Figure 8:**
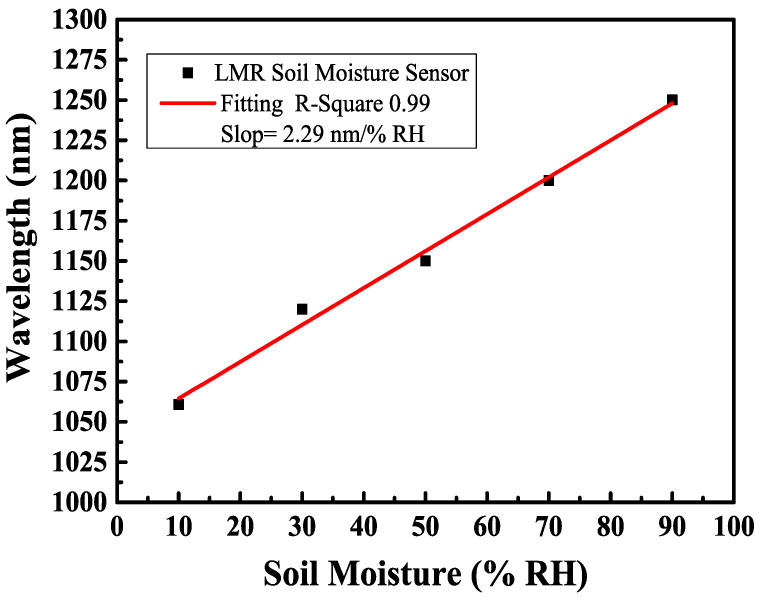
Linear fitting of sensitivity of single-layer SnO_2_ film coated on D-shaped fiber.

These spectral attenuation positions also depend on the refractive index of the surrounding medium (i.e., the external refractive index). This fact enables the application of LMR sensing structures as liquid refractometers [43,44]. Thus, LMR dips shift to longer wavelengths as the soil moisture contents increase. Figure 8 displays a linear fitting curve diagram of the sensitivity of the LMR multimode D-shaped fiber coated with the SnO_2_ thin film. The film thickness of the SnO_2_ thin film was 450 nm, and the maximum sensitivity was 2.29 nm/%RH when the relative humidity varied between 10%RH and 90%RH. The sensing mechanism, structural design, and sensing performance of humidity sensors based on the fiber optic technique in the most recent five years have been reviewed. To better and clearly understand the sensing performance of different humidity sensors, their main parameters are summarized in Table 1, including sensor structure, the selection of functional materials, dynamic range, and sensitivity. The results show that the proposed LMR-based fiber optic sensor exhibits higher sensitivity in measuring soil relative humidity in the range of 10%RH to 90%RH than that reported in the literature. In order to achieve higher sensitivity, the use of optimization technology for D-shaped fibers and sensitive material coating thickness has become an important approach to improving sensing performance.

### 4.2. Response Time Test

As mentioned before, the position of the resonant wavelength of LMR changes with the relative humidity of the external medium, and the refractive index of the external medium and SnO_2_ thin film coating also change accordingly. The change in the refractive index of the coating can be explained by the adsorbed water on the oxide surface within the SnO_2_ structure. The goal of the LMR sensor is to obtain the relative humidity of soil through an electronic Arduino sensor while collecting infrared transmission spectra. Then, the relationship between the wavelength positions of RH and LMR can be established.

**Figure 9:**
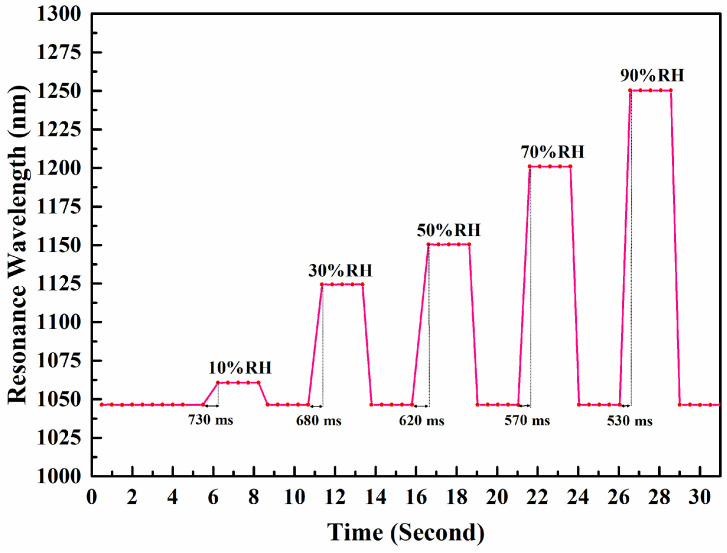
A plot of the resonant wavelength of the LMR sensor as a function of time as soil moisture changes from 10% to 90%RH. The pink line is the measured data and the grey line marks the response time.

A fast response time is important when a sensor is responsible for measuring a rapidly changing dynamic relative humidity (RH). This study evaluates the response time of the proposed LMR sensor by burying the fiber optic sensor in soil with rapidly changing relative humidity. We performed experiments to obtain a plot of the LMR sensor resonant wavelength as a function of time under the condition of varying soil moisture in the range of 10% to 90%RH (at a temperature of 25 °C). Figure 9 shows a plot of the resonant wavelength of the LMR sensor as a function of time. The response time of the LMR sensor decreased slightly with increasing relative humidity, from 730 ms to 530 ms. The average response time is 626 ms.

**Figure 10:**
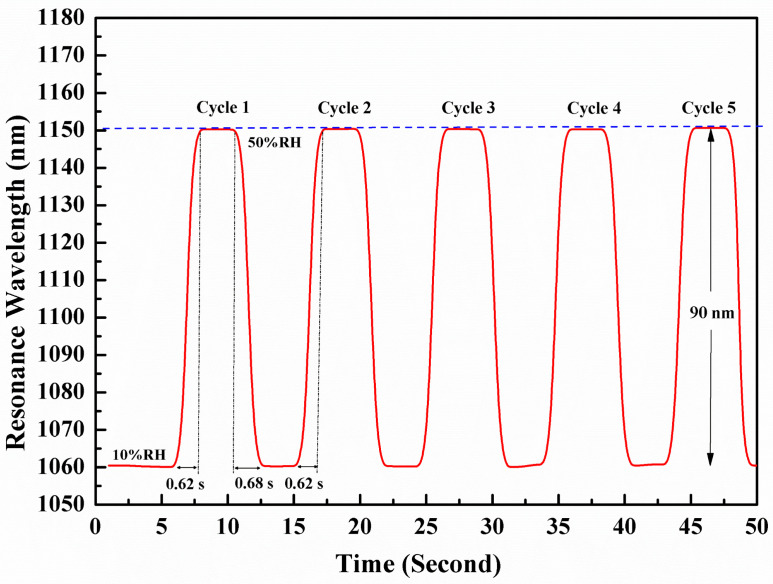
The response time of the LMR sensor to soil moisture switching from 10% RH to 50% RH under five soil moisture cycles. The red line is the measurement data, the purple line represents the 1150 nm scale, and the gray line represents the response time.

Fiber optic sensors have the advantage of fast response times compared to electrical relative humidity sensors. Five cycles of the response and recovery curves demonstrate that the proposed sensor has good repeatability in continuous switching measurement when the relative humidity changes from 10% to 50%, as shown in Figure 10. In the response time test, two humidity conditions of 10% RH and 50% RH were set, and the moving platform was used to move quickly in both the 10% and 50% RH environments, with the moving time controlled within 1 s. We kept the temperature at 25 °C and quickly recorded the data using a personal computer. The response time of the LMR sensor under five soil moisture cycles is 0.62 s, and the soil moisture switches from 10% to 50% RH. When RH changes from 50% to 10%RH, the recovery time is 0.68 s. These results indicate that the response of the LMR sensor to soil moisture switching from 10% RH to 50% RH, as well as the response under five soil moisture cycles, can demonstrate the effectiveness and reproducibility of the proposed sensor. Our method exhibits a fast response time and rapid recovery period, which are significantly improved compared to those of some RH fiber optic sensors reported on in the literature [45].

## 5. Conclusions

Fiber optic sensors based on the LMR effect have attracted widespread research and development interest due to their unique advantages such as high sensitivity and label-free measurement. In this work, we developed a high-sensitivity fiber optic soil moisture sensor based on LMR using a thin film coating and side-polished fiber optic technology. A single-layer tin dioxide (SnO_2_) thin film was used as the humidity sensing material and coated on a D-shaped multimode optical fiber. For the performance of relative humidity fiber optic sensors, we compared various fiber optic structures with different coating materials. The proposed LMR-based D-shaped fiber optic soil moisture sensor was coated with a single layer of tin dioxide and had a sensitivity of 2.29 nm/%RH. The results show that the D-shaped multimode fiber with a remaining thickness of 93 had has the maximum sensitivity to the soil moisture range of 10%RH~90%RH. The proposed LMR-based optical fiber sensor shows a fast response time and good reproducibility, and it also presents a larger dynamic range and higher sensitivity than other fiber optic sensors.

In summary, fiber optic soil moisture sensors based on D-shaped multimode fibers and thin film coating technology have shown promising prospects for high-sensitivity measurements. The combination of D-shaped geometry, multimode fibers, and the thin film coating technique can provide a robust platform for soil moisture detection. Further research could focus on optimizing coating materials and fiber geometry to enhance sensitivity and selectivity for soil moisture applications.

## Figures and Tables

**Table 1:** Performance comparison of humidity fiber sensors in past five years.

Structure	Materials	Range (%RH)	Max. Sensitivity	References
LMR-based MMF	Graphene oxide	20–90%RH	0.612 nm/%RH	[20]
LPG array	Silica nanoparticle	45–95%RH	0.53 nm/%RH	[21]
Fiber Bragg grating probe	PMMA	0–40%RH	0.018 nm/%RH	[22]
Fiber Bragg grating	Polyimide	11–97%RH	2.0 pm/%RH	[24]
Fabry–Perot based	PNIPAM	45–75%RH	1.634 nm/%RH	[25]
Localized SPR	Au nanosphere	60–90%RH	0.634 nm/%RH	[26]
LMR-based MMF	SnO_2_ thin film	10–90%RH	2.29 nm/%RH	This work

## Data Availability

No new data were created or analyzed in this study. Data sharing is not applicable to this article.
